# Supplementary material for: Recent warming reduces the reproductive advantage of large size and contributes to evolutionary downsizing in nature
Source: Proc Biol Sci. 2020 Jun 3;287(1928):20200608. doi: 10.1098/rspb.2020.0608 (PMC7341922; doi:10.1098/rspb.2020.0608)
Supplement: Fryxell ESM Tables and Figures [file rspb20200608supp1.pdf]

Electronic Supplementary Material for Proceedings of the Royal Society B

Recent warming reduces the reproductive advantage of large size and contributes to evolutionary downsizing in nature (DOI: 10.1098/rspb.2020.0608)

David C. Fryxell*[1,2]

Alexander N. Hoover[2]

Daniel A. Alvarez[2]

Finn J. Arnesen[2]

Javiera N. Benavente[1]

Emma R. Moffett[1]

Michael T. Kinnison[3]

Kevin S. Simon[†1]

Eric P. Palkovacs[†2]

* corresponding author: dfry901@aucklanduni.ac.nz, orcid: 0000-0003-4543-4809

[†] co-senior authorship

Affiliations

1. School of Environment, University of Auckland, Auckland 1010, New Zealand
2. Department of Ecology and Evolutionary Biology, University of California, Santa Cruz 95060, California, USA
3. School of Biology and Ecology, University of Maine, Orono, Maine, USA

Table S1: Focal site characteristics, ordered by mean site temperature from the coolest site to the warmest site (from data in Fig 1). We approximated site area by drawing Google Earth polygons and calculating its area at http://earthpoint.us/shapes.aspx. We measured pH with an Oakton PT Testr, and measured dissolved oxygen and specific conductance with a YSI Pro DO. We measured water PO_4 and NO_x concentrations from previously filtered (0.7 µm) then frozen site water using standard manufacturer methods on a LaChat flow injection analyzer. We determined pelagic chlorophyll a concentration after acetone extraction using the non-acid module on a Turner Designs Trilogy Fluorometer. Bullfrogs are nonnative American bullfrogs (*Lithobates catesbeianus*), crayfish are nonnative red swamp crayfish (*Procambarus clarkii*), and pupfish are native and endangered Owens pupfish (*Cyprinodon radiosus*). We gathered these data on the same dates for each characteristic, albeit on different dates across characteristics. Because these are stable, spring-fed systems, we expect these point-estimates to reflect largely consistent variation among sites.

Spring name	Name code	Tmean (°C)	Tvar (SD)	Max depth (m)	Site area (m²)	pH	Specific cond. (mS/cm)	Diss. oxygen (mg/L)	[PO4] (ppm P)	[NOx] (ppm N)	Chlorophyll a (µg/L)	Spring-head location	Barrier - purpose	Other large animals
Northeast Spring	NE	18.85	0.835	1.7	101	8.3	0.36	8.26	0.03	0.68	0.24	In pond	Manmade - unknown	Crayfish
BLM Spring	BLM	21.06	1.700	1.7	886	8.2	0.47	6.98	0.01	0.33	0.65	In pond	Manmade - pupfish	Pupfish, Crayfish
Artesian Well	AW	23.66	0.627	1.7	350	7.4	0.45	4.49	0.03	0.17	1.66	In pond	Manmade - cattle	Crayfish
Small Artesian Well	LAW	24.84	0.357	2.0	75	7.4	0.45	6.26	0.02	0.02	1.95	In pond	Manmade - cattle	None
Warm Springs	WSU	27.00	1.230	2.0	435	7.8	0.51	7.91	0.01	0.11	0.40	Just upstream	Manmade - pupfish	Crayfish, Bullfrogs
Keough Hot Ditch	K2	31.57	2.124	1.2	228	8.4	0.86	5.31	0.01	0.16	0.36	Upstream	Natural - NA	None
Little Hot Creek	LHC	33.31	2.263	1.0	127	8.2	2.04	6.11	0.05	0.04	1.81	Upstream	Natural - NA	None
**Cor(Tmean, Y)**		1	0.66	-0.67	-0.31	0.18	0.79	-0.42	0.24	-0.72	0.26			

Table S2: Sampling dates, sample sizes (of gravid females), and temperatures for each wild fish site x sampling occasion combination.

	Site Code	Temp. (°C)	Collection Date	Sample Size
**Spring**	NE	18.85	21 March 2015	23
	BLM	21.06	21 March 2015	9
	AW	23.66	22 March 2015	40
	LAW	24.84	22 March 2015	NA*
	WSU	27.00	23 March 2015	31
	K2	31.57	22 March 2015	33
	LHC	33.31	21 March 2015	10
**Summer**	NE	18.85	5 June 2014	39
	BLM	21.06	5 June 2014	41
	AW	23.66	5 June 2014	32
	LAW	24.84	6 June 2014	18
	WSU	27.00	6 September 2018	3
	WSU	27.00	5 June 2014	35
	K2	31.57	6 June 2014	28
	K2	31.57	6 September 2018	25
	LHC	33.31	6 June 2014	35
**Spring total**				**146**
**Summer total**				**256**

* predatory largemouth bass introduced before this sampling date

Table S3: F1 fish recovered by date, and the number that were dissected and gravid and thus included in generalized linear modelling (i.e. “# gravid, dissected”). Based on these temporal patterns, we estimate that at least 12 individuals contributed offspring to the F1 generation, because offspring were found on at least 12 days, and female mosquitofish release entire broods in single bouts over a short time period. This is a minimum estimate because multiple females likely gave birth on the same day. Moreover, female mosquitofish are likely to receive sperm from multiple males, which suggests a significant genetic contribution of many individuals from each population to the F1 generation.

	**Population**						
**Date**	NE	BLM	AW	WSU	K2	LHC	**Daily totals**
19 March 2018			15			2	17
20 March 2018		5					5
21 March 2018	30	7	4			16	57
22 March 2018	45	35			21		101
23 March 2018	21	5			1		27
24 March 2018	32						32
25 March 2018	45		1	10	21	4	81
26 March 2018	60	2	16	15	14	2	109
27 March 2018	13	11	25	13	22	3	87
28 March 2018	3		34		2	3	42
29 March 2018	11	3	30		5	1	50
30 March 2018			6	3	7	6	22
31 March 2018	4		8		4	30	46
1 April 2018		1	18	1	11	3	34
2 April 2018		11	13	11			35
3 April 2018	1	20	1				22
4 April 2018		14	10	1		6	31
5 April 2018					5	20	25
6 April 2018		3				9	12
7 April 2018						6	6
8 April 2018			25	17			42
9 April 2018		1	5	3		13	22
10 April 2018	40	44		3		4	91
11 April 2018	42	24	46	1	2	16	131
12 April 2018	8	13	20				41
13 April 2018	18	5	13				36
14 April 2018						4	4

15 April 2018	14	3	2	10		5	34
**# produced:**	387	207	292	88	115	153	1242
**# dissected, gravid:**	35	24	31	25	24	29	168

Table S4: Comparison of the OLS model (using the lm() function in R) predicting juvenile growth to three robust linear regression methods that are meant to deal with violations of OLS model assumptions. The rlm() function requires the 'MASS' package. The lmrob() function requires the 'robustbase' package. Tables were generated using the summary() function for each model. Compared to the robust methods, the OLS method led to stronger effects of rearing temperature (especially the first-order term) and weaker effects of source temperature. Thus, we decided to use the OLS results in the main text as a conservative estimate of the strength of source temperature effects relative to rearing temperature effects.

Method	Coefficient	Estimate	Std. Error	t-value	p-value
lm	intercept	0.3835	0.0164	23.402	< 2E-16
	poly(reartemp, 2)	3.5366	0.1175	30.110	< 2E-16
	poly (reartemp, 1)	-0.7316	0.1179	-6.204	8.62E-10
	sourcetemp	-0.0029	0.0006	-4.586	5.21E-06
rlm, Huber weights	intercept	0.3935	0.0153	25.681	NA
	poly(reartemp, 2)	3.6378	0.1098	33.123	NA
	poly (reartemp, 1)	-0.6349	0.1103	-5.758	NA
	sourcetemp	-0.0032	0.0006	-5.461	NA
rlm, bisquare weights	intercept	0.3951	0.0153	25.766	NA
	poly(reartemp, 2)	3.6773	0.1099	33.453	NA
	poly (reartemp, 1)	-0.5853	0.1104	-5.303	NA
	sourcetemp	-0.0033	0.0006	-5.533	NA
lmrob	intercept	0.3949	0.0152	25.952	< 2E-16
	poly(reartemp, 2)	3.6751	0.1063	34.580	< 2E-16
	poly (reartemp, 1)	-0.5868	0.1061	-5.529	4.30E-08
	sourcetemp	-0.0033	0.0006	-5.637	2.36E-08

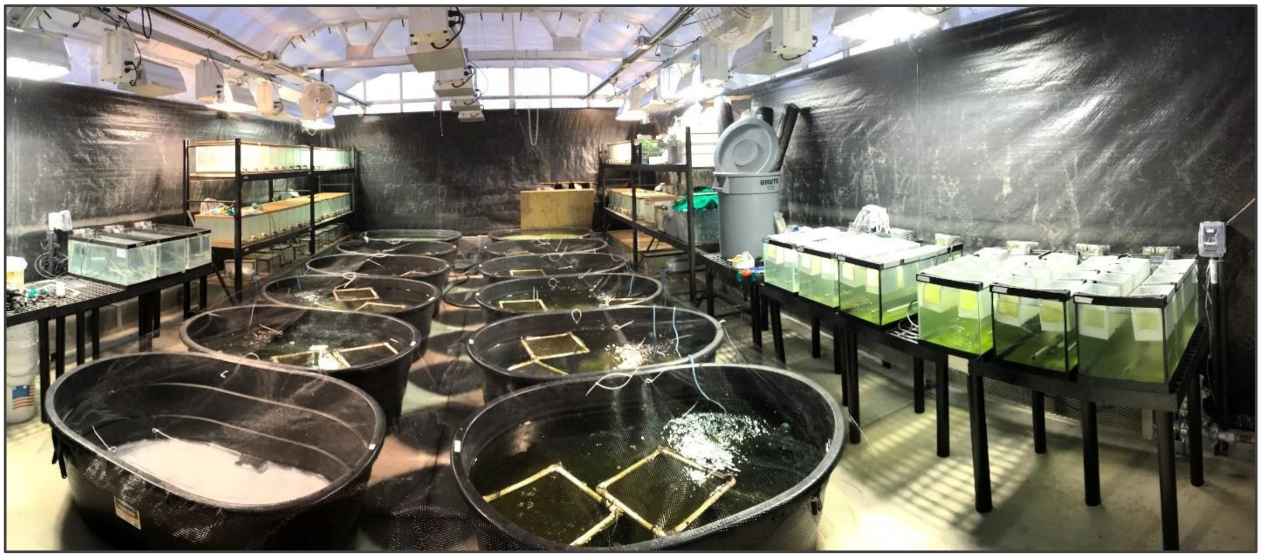

Fig S1: Controlled environment greenhouse where F0 to F1 common-rearing took place. Shown are the N=6 568 L black plastic tanks with fry retention devices (floating white squares) and N=9 57 L glass aquaria where newborn F1 fish were temporarily reared in white plastic “fry baskets” (dimensions 10 x 10 x 15 cm^3).

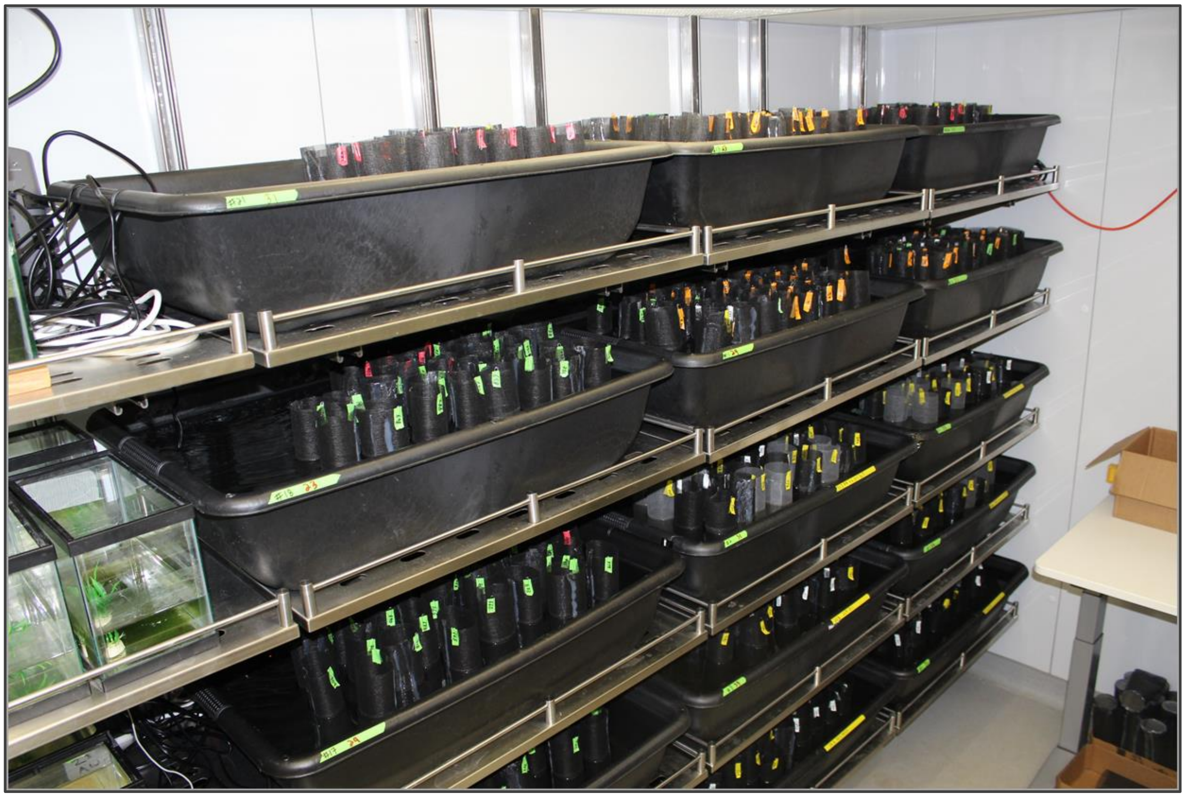

Fig S2: One of two controlled environment rooms where F2 fish were reared. Shown are the black 100 L plastic tanks. Individual fish were reared in black mesh containers (Fig S3), sunk into these tanks.

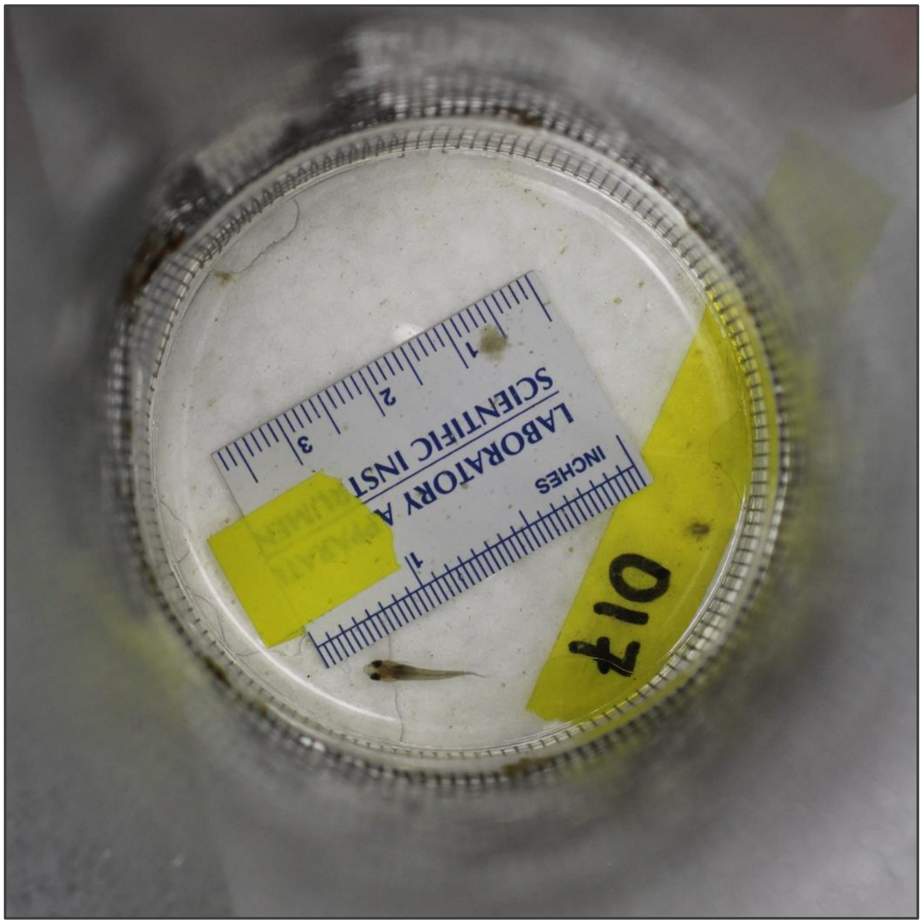

Fig S3: Example top-down photo of a 15-day old fish in its individual rearing container. These photos were taken at age 0 days and at age 15 days to calculate growth rates for each fish.

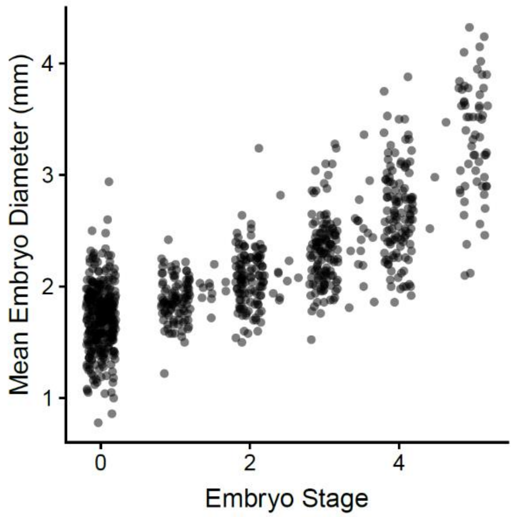

Fig S4: Embryo size (diameter) versus embryo stage across all fish studied (wild & F1 combined), indicating that life history traits likely depend on the stage of embryonic development. Points are jittered along the x-axis.

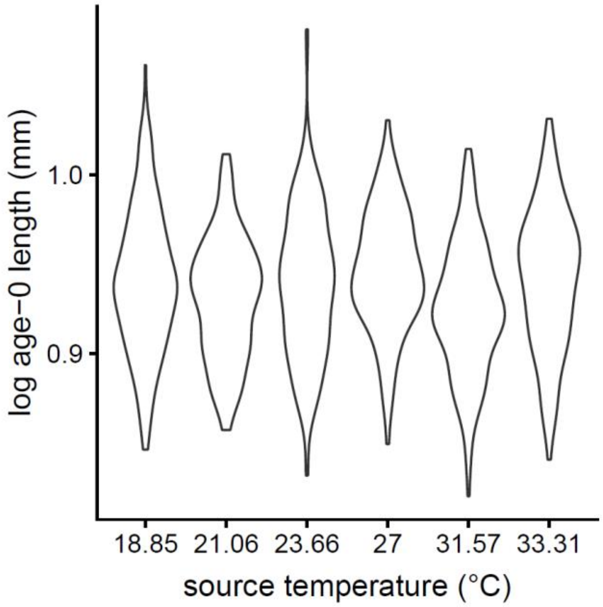

Fig S5: Violin plots of the total length of newborn F2 fish (overall n=1137) from each of the six source populations, measured the day of parturition. Source temperature did not predict $\log_{10}$ newborn length ($p = 0.292$, $R^2 < 0.01$).

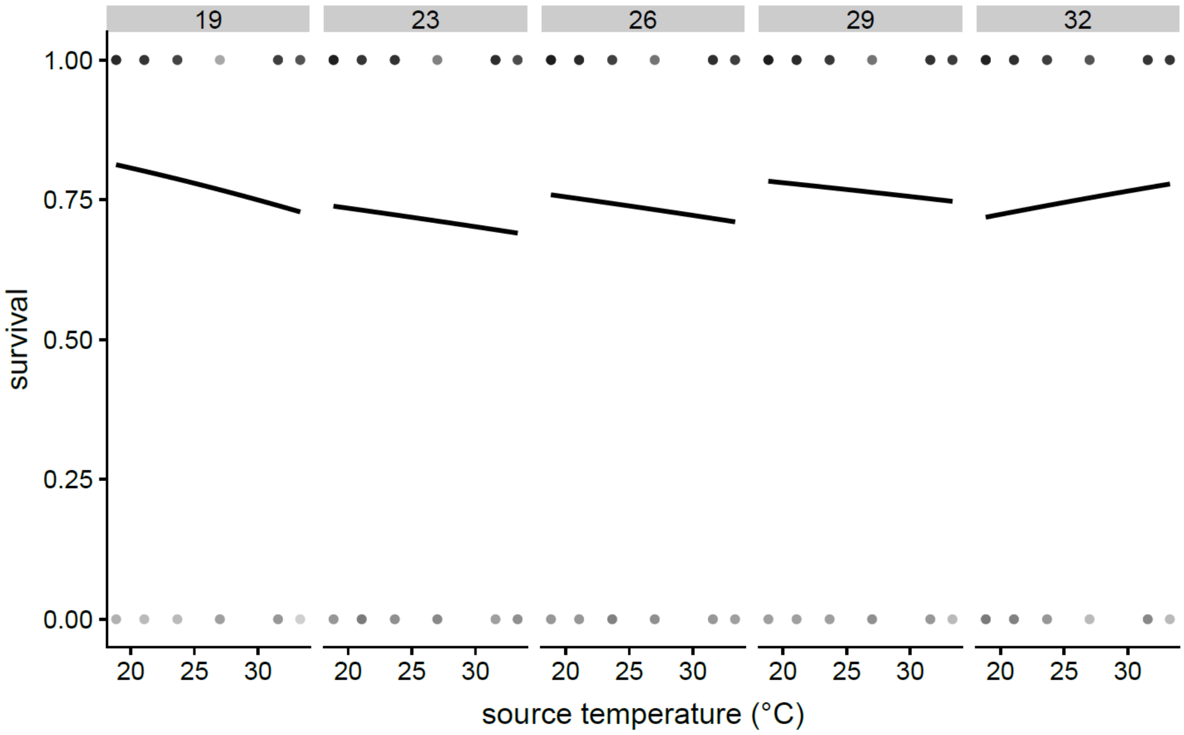

Fig S6: F2 fish survival from age 0 to age 15 days. At each rearing temperature (faceting), survival was roughly 75%. Survival did not significantly differ by source temperature, rearing temperature (as a factor), or its interaction (all $p > 0.401$). Lines are from a generalized linear model with a binomial error distribution.

Appendix S1. Life history dissection protocol

Gambusia Life History Dissection Protocol Fryxell updated 7/9/2018

1) Remove female fish from sample container. Leave the fish in ethanol until prepared to execute all the steps below.
2) Blot fish dry with kimwipe, immediately weigh the fish using analytical balance (g)
3) Take 3 replicate total length measurement w digital calipers (mm)
4) Cut open entire body cavity on left side of fish (square cut exposing all entrails)
5) Remove entire gonad, ideally in one piece.
6) Weigh gonads (blot dry if necessary) (g)
7) Under dissecting scope (from here out) isolate the embryos by carefully picking them apart. Avoid disrupting the natural shape of the embryos.
8) Count the number of embryos, note their average stage (all should be approximately at the same stage; see diagram from Reznick 1981*, but use whole number assignments 0-5 except for F1 fish for which ½ increment assignments can be made) and then using max magnification (45x) and a stage micrometer measure the length of the longest dimension of 5 haphazardly (but intact; i.e. not inadvertently bent or destroyed while picking them apart) chosen embryos.
9) Weigh all embryos together (without other gonad tissue).
10) Check to ensure you've recorded all the data. Note any abnormalities about the fish or about the way it was measured in the "notes" column.
11) Discard fish parts in the trash and proceed to the next fish.
12) When done for the day, clean up tools, equipment, and benchtop area. Look over data sheet, checking for errors. Save the data sheet and close it.

*Reznick, D. (1981). "Grandfather effects": The genetics of offspring size in the mosquitofish *Gambusia affinis*. Evolution, 35, 941-953.
